# Identification of HOX signatures contributing to oral cancer phenotype

**DOI:** 10.1038/s41598-022-14412-6

**Published:** 2022-06-16

**Authors:** Kanaka Sai Ram Padam, Richard Morgan, Keith Hunter, Sanjiban Chakrabarty, Naveena A. N. Kumar, Raghu Radhakrishnan

**Affiliations:** 1 Department of Cell and Molecular Biology, Manipal School of Life Sciences, Manipal Academy of Higher Education, Manipal, Karnataka 576104 India; 2 School of Biomedical Sciences, University of West London, London, W5 5RF UK; 3 Academic Unit of Oral and Maxillofacial Medicine and Pathology, School of Clinical Dentistry, University of Sheffield, Sheffield, S10 2TA UK; 4 Department of Surgical Oncology, Kasturba Medical College and Hospital, Manipal Academy of Higher Education, Manipal, Karnataka 576104 India; 5 Department of Oral Pathology, Manipal College of Dental Sciences, Manipal, Manipal Academy of Higher Education, Manipal, 576104 India

**Keywords:** Cancer, Computational biology and bioinformatics, Drug discovery

## Abstract

The role of evolutionarily conserved homeobox-containing *HOX* genes as transcriptional regulators in the developmental specification of organisms is well known. The contribution of *HOX* genes involvement in oral cancer phenotype has yet to be fully ascertained. TCGA-HNSC HTSeq-counts and clinical data were retrieved from the GDC portal for oral cavity neoplasms. GEO datasets (GSE72627, GSE30784, GSE37991) were accessed and analyzed using GEO2R. Differential *HOX* gene expression was profiled using the DESeq2 R package with a log2 fold change cut-off (− 1 and + 1) and Benjamini–Hochberg *p*-adjusted value at ≤ 0.01. Gene set over-representation analysis and semantic analysis associated with the disease ontology was performed using the ClusterProfiler R package, and pathway over-representation analysis was performed using IMPaLa. HOX protein interaction network was constructed using the Pathfind R package. *HOX* phenotype associations were performed using Mammalian Phenotype Ontology, Human Phenotype Ontology, PhenGenI associations, Jensen tissues, and OMIM entries. Drug connectivity mapping was carried out with Dr. Insight R package. *HOXA2* was upregulated in oral dysplasia but silenced during tumor progression. Loss of *HOXB2* expression was consistent in the potentially malignant oral lesions as well as in the primary tumor. *HOXA7, HOXA10, HOXB7, HOXC6, HOXC10, HOXD10,* and *HOXD11* were consistently upregulated from premalignancy to malignancy and were notably associated with risk factors. Overrepresentation analysis suggested *HOXA10* was involved in the transcriptional misregulation contributing to the oral cancer phenotype. *HOX* genes subnetwork analysis showed crucial interactions with cell cycle regulators, growth responsive elements, and proto-oncogenes. Phenotype associations specific to the oral region involving *HOX* genes provide intrinsic cues to tumor development. The 5′ *HOX* genes were aberrantly upregulated during oral carcinogenesis reflecting their posterior prevalence.

## Introduction

*HOX* genes are a subset of homeobox genes, which function as transcriptional regulators specifying the anteroposterior (A-P) axis of the animal body plane involved in the developmental organization of structures or organs leading to the morphological changes^1^. There are a total of 39 *HOX* genes, which are segregated into four clusters *HOXA* (7p15), *HOXB* (17q21.2), *HOXC* (12q13), and *HOXD* (2q31)^2^. The clustered topology of *HOX* genes in the mammalian genome corresponds to their magnitude and the order of expression in a collinear fashion which is either regulated or coordinated^3,4^. The expression of *HOX* genes is coordinated from 3′ to 5′ temporally corresponding to their position along the A-P axis during vertebrate development. The *HOX* genes situated towards the 3′ are termed the anterior *HOX* genes whereas those situated towards the 5′ are termed the posterior *HOX* genes^5,6^.

Patterns of disruption of *HOX* gene expression due to temporospatial deregulation were first described by Abate-Shen^7^. The differential expression in tumor tissues has shown to be associated with a perturbation of normal organogenesis and differentiation. Epigenetic interplay involved in the process of gene transcription promoting neoplastic transformation may be the underlying factor leading to altered expression of *HOX* genes in tumor tissues^7^. During the disease state, the 5′ positioned *HOX* genes show a dominant phenotype compared to the 3′ and are thus termed the *HOX* genes of posterior prevalence. These observations are evident in oesophageal squamous cell carcinoma where the normal foregut shows 3′ *HOX* dominated expression but is lost in the tumor tissues, which exhibits a dominant expression of 5′ *HOX* genes^8^.

**Figure 1:**
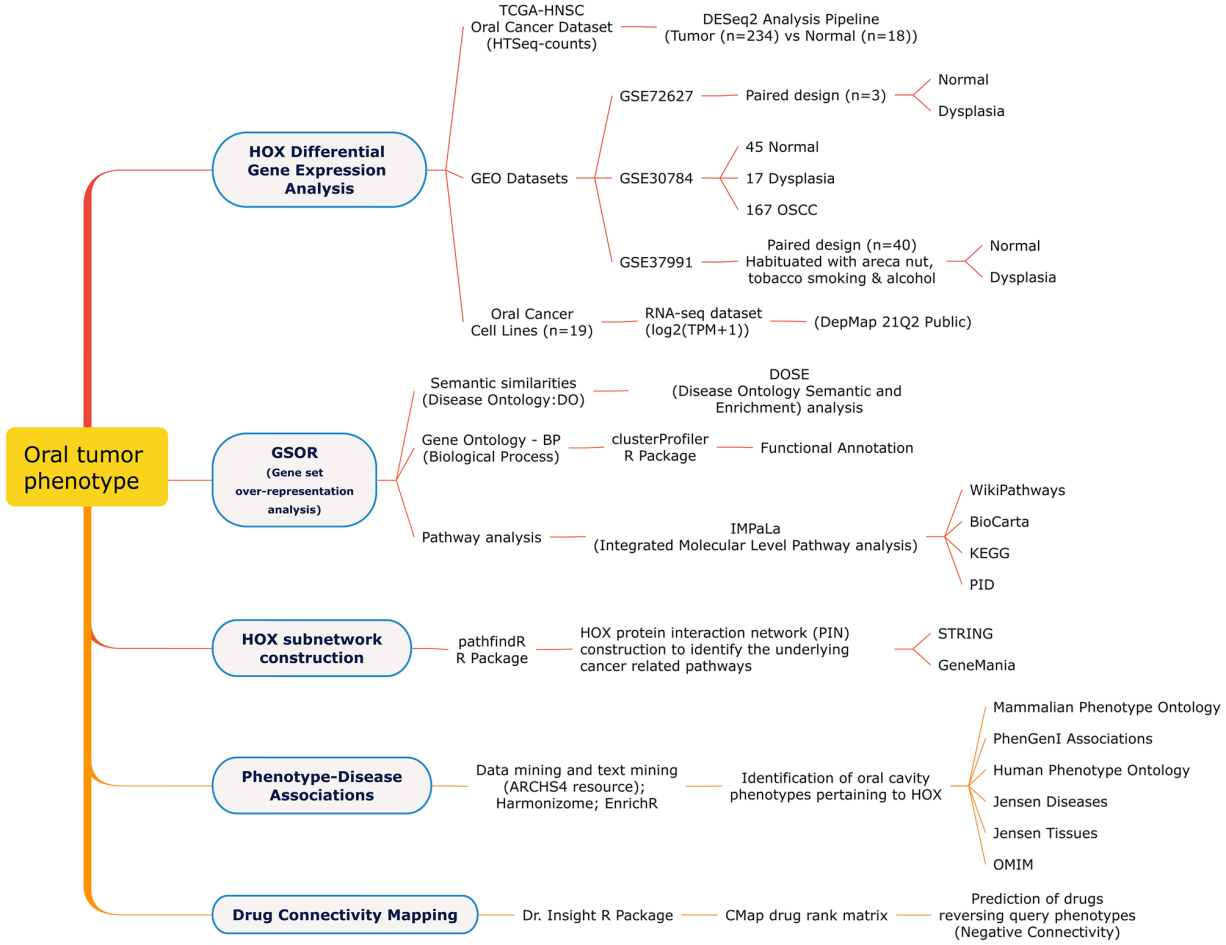
Schematic workflow employed to identify the *HOX* genes involved with oral tumor phenotype.

Several studies have shown that deregulated *HOX* expression in oral cancer^6,7,9–12^ is either due to loss of tissue specificity or epigenetically-mediated loss of function^6,7,13^. The regulatory role of *HOX* genes in determining tumor characteristics and factors contributing to oral cancer phenotype, in particular, is the basis of this study. In this paper, the crucial interactions of *HOX* subnetworks were computationally analyzed to understand the role of deregulated *HOX* genes in transition to the oral tumor phenotype and identify potential therapeutic targets. The workflow employed is illustrated in Fig. 1.

## Methodology

### Data acquisition

Publicly available gene expression datasets deposited in the Gene Expression Omnibus (GEO) (https://www.ncbi.nlm.nih.gov/geo/) were accessed to query curated gene expression profiles. The following search terms “(oral cancer) OR dysplasia OR leukoplakia AND/OR transcriptome AND (areca nut/betel quid) OR smoking OR alcohol OR Tobacco” were used. The GEO datasets, GSE72627 consisted of paired normal and tumor samples (n = 3) derived from three patients^14^. GSE30784 included 45 normal, 17 dysplasia, and 167 oral cancer samples^15^ and GSE37991 consisted of paired normal and oral tumor samples (n = 40) associated with risk factors such as smoking tobacco, drinking alcohol and chewing areca nut^16^.

The transcriptome profile of oral cancer-causing genomic alterations, cataloged in The Cancer Genome Atlas – Head and Neck Squamous cell Carcinoma (TCGA-HNSC)^17^ derived oral cancer datasets consisted of 18 normal and 234 primary tumor cases. HTSeq-counts data was accessed and downloaded from Genomic Data Commons (GDC) repository (https://portal.gdc.cancer.gov/). The 10th revision of the International Classification of Diseases (ICD-10) was used to define cancer of the oral cavity, which included cancer of the lip (C00.9), border of the tongue (C02.1), ventral surface of the tongue (C02.2), unspecified parts of the tongue (C02.9), upper gum (C03.0), lower gum (C03.1), unspecified parts of the gum (C03.9), the anterior floor of the mouth (C04.0), floor of the mouth (C04.9), cheek mucosa (C06.0), retromolar area (C06.2) and unspecified parts of the mouth (C06.9). Patients with insufficient or missing clinicopathological data were excluded from the subsequent analysis.

### Identification of differential expression of HOX genes

The GSE30784 and GSE37991 oral cancer datasets were analyzed for profiling the differential *HOX* gene expression using the interactive web tool GEO2R, (https://www.ncbi.nlm.nih.gov/geo/geo2r/) to compare two or more groups of samples^18^. The transcriptome profile (GSE72627) of paired normal and moderate dysplasia samples (n = 3) derived from three patients were analyzed using “ggppubr” for data visualization in R. The paired analysis specific to *HOX* genes was illustrated using “ggboxplot” and “ggpaired” functions. Genome-wide annotation for the HTseq-counts data derived from TCGA oral cancer cohort was performed using org.HS.eg.db, Bioconductor version: Release (3.13) and R package (v4.1) (R Core Team, 2020. R: A language and environment for statistical computing. R Foundation for Statistical Computing, Vienna, Austria. https://www.r-project.org/). Quality control, normalization and differential expression of the *HOX* genes in the sample cohorts were performed using the DESeq2 package^19^, which uses negative binomial distribution to model RNA-seq counts data. A log2 fold change of + 1 and − 1 was used as a threshold with 1% FDR corrected with the Benjamini–Hochberg procedure to determine the differentially expressed *HOX* genes. An adjusted *p*-value of ≤ 0.01 was considered to be statistically significant. Data visualization was carried out using pheatmap and EnhancedVolcano R packages.

### Oral cancer cell line analysis

The RNA-seq datasets (DepMap, Broad (2021): DepMap 21Q2 Public) were accessed and downloaded from the Dependency Map portal (https://depmap.org/portal/) to analyze the expression patterns and regulation of *HOX* genes in oral cancer-derived cell lines. A total of 19 oral cancer cell lines were screened for the expression patterns of *HOX* genes. An illustrative heatmap was constructed to analyse the cell line-specific *HOX* gene expression pattern using the pheatmap R package. The cell line data specific to each *HOX* gene were pooled together to calculate the average expression using the ggplot2 R package. The cell lines with missing clinical characteristics were excluded from the subsequent analysis.

### Gene set over-representation analysis

Over-representation analysis (ORA) was performed to identify the biological process and the semantic similarities of disease ontology (DO) associated with the significant differentially expressed *HOX* genes in oral cancer using clusterProfiler^20,21^. The categories relevant to the study were filtered (adjusted *p*-value < 0.05) and depicted as a heatplot. Further, the pathway over-representation analysis was performed using IMPaLa (Integrated Molecular Pathway Level analysis)^22^ to identify the list of pathways involved. A *p*-value of < 0.05 was considered statistically significant.

### HOX subnetwork analysis

Underlying disease states were further predicted by constructing a protein interaction network (PIN). Anticipated protein–protein interaction (PPI) information was complemented using STRING^23^ and genes were prioritized for functional assays using GeneMania^24^. To identify the distinct active subnetwork(s) associated with the *HOX* genes, the pathfindR^25^ package was used. Further, the subnetwork-oriented pathway enrichment analysis was performed using KEGG^26–28^ to exploit the disease alterations by this subset of genes in interaction with *HOX* genes using KEGG pathways.

### HOX phenotypic and disease associations

The differentially expressed *HOX* genes were further processed downstream to predict their phenotype associations with oral cancer development. Data and text mining was performed for integration of the query *HOX* genes to identify their association with phenotypes accessing the Mammalian Phenotype Ontology^29^, Human Phenotype Ontology (HPO)^30^, PhenGenI associations, Jensen diseases^31^, Jensen tissues^32^ and OMIM^33^ using Enrichr^34,35^. It was further supplemented with harmonizome^36^, an integrated analysis tool about genes and proteins for massive mining of the publicly available RNA-seq data, ARCHS4^37^ resource. The biomolecular-phenotype network of association of the *HOX* genes and phenotypes specific to the oral cavity has been illustrated using Cytoscape^38^.

### Drug connectivity mapping

Drug connectivity mapping was carried out using CMap^39^ drug rank matrix dataset and the query dataset matrix containing significantly differentially expressed *HOX* genes with a t-test statistic score computed using DESeq2^19^. Drug identification analysis was performed in an attempt to identify the drugs that could reverse the query disease phenotype (negative connectivity) following the perturbation using the Dr. Insight R package^40^. The query results were adjusted to and sorted by a *p*-value of < 0.05 as a measure of significance and illustrated using Cytoscape^38^.

### Ethics approval

Data was freely available from the public domains which are properly anonymized and informed consent was obtained at the time of data collection and did not require any ethical approval.

## Results

### Differentially expressed HOX genes show posterior prevalence in oral cancer

**Figure 2:**
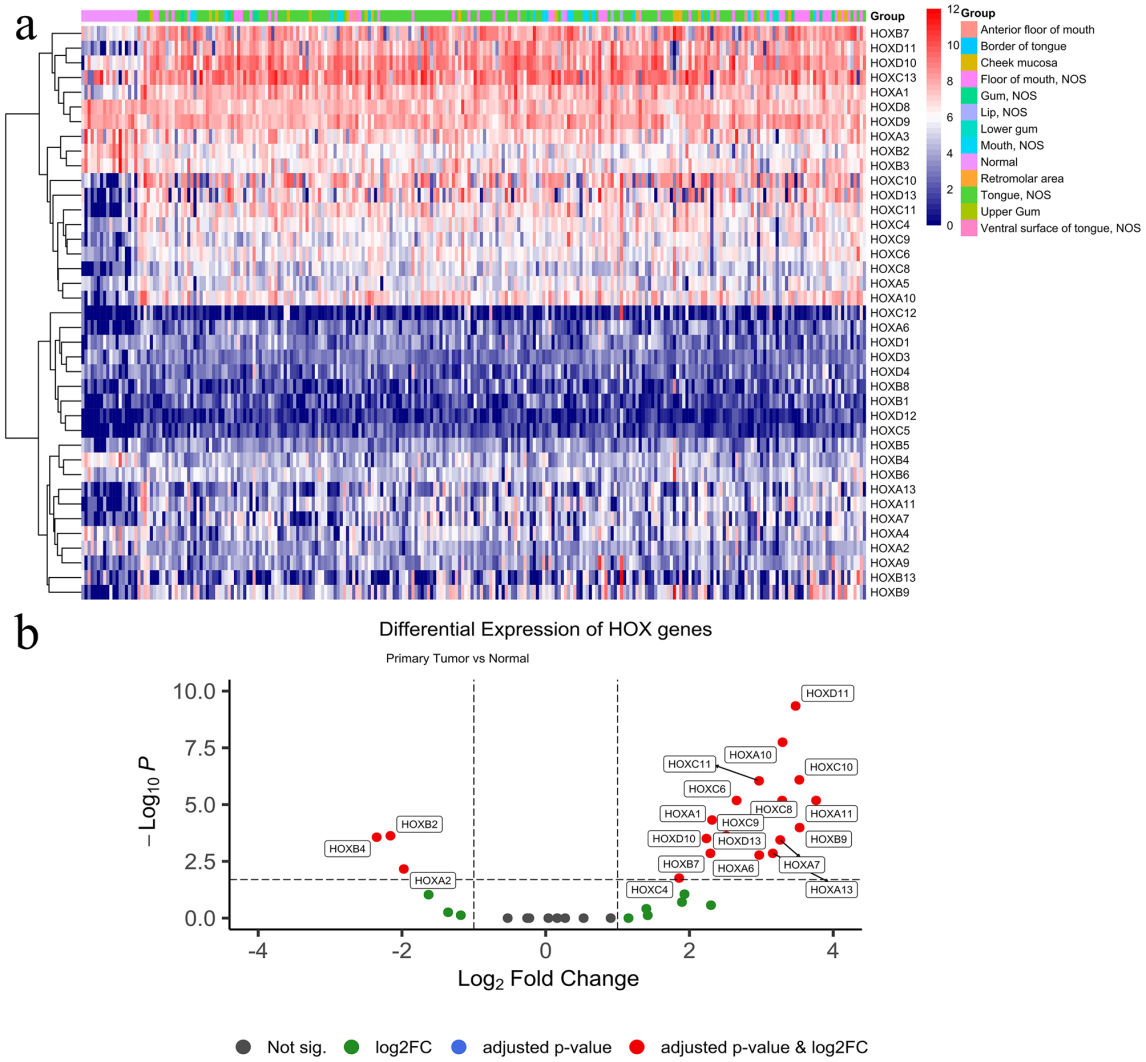
(**a**–**b**): (**a**) Illustrative heatmap of *HOX* gene expressions in a panel of normal (n = 18) and tumor (n = 234) specimens analyzed from the TCGA-HNSC derived oral cancer datasets categorized based on their subsites. (**b**) Differentially expressed *HOX* genes in primary tumors compared to the normal samples were identified using DESeq2 with a log2FC cut-off (− 1 and + 1) factoring 1% FDR to minimize false positives. Results are depicted as a volcano plot.

**Figure 3:**
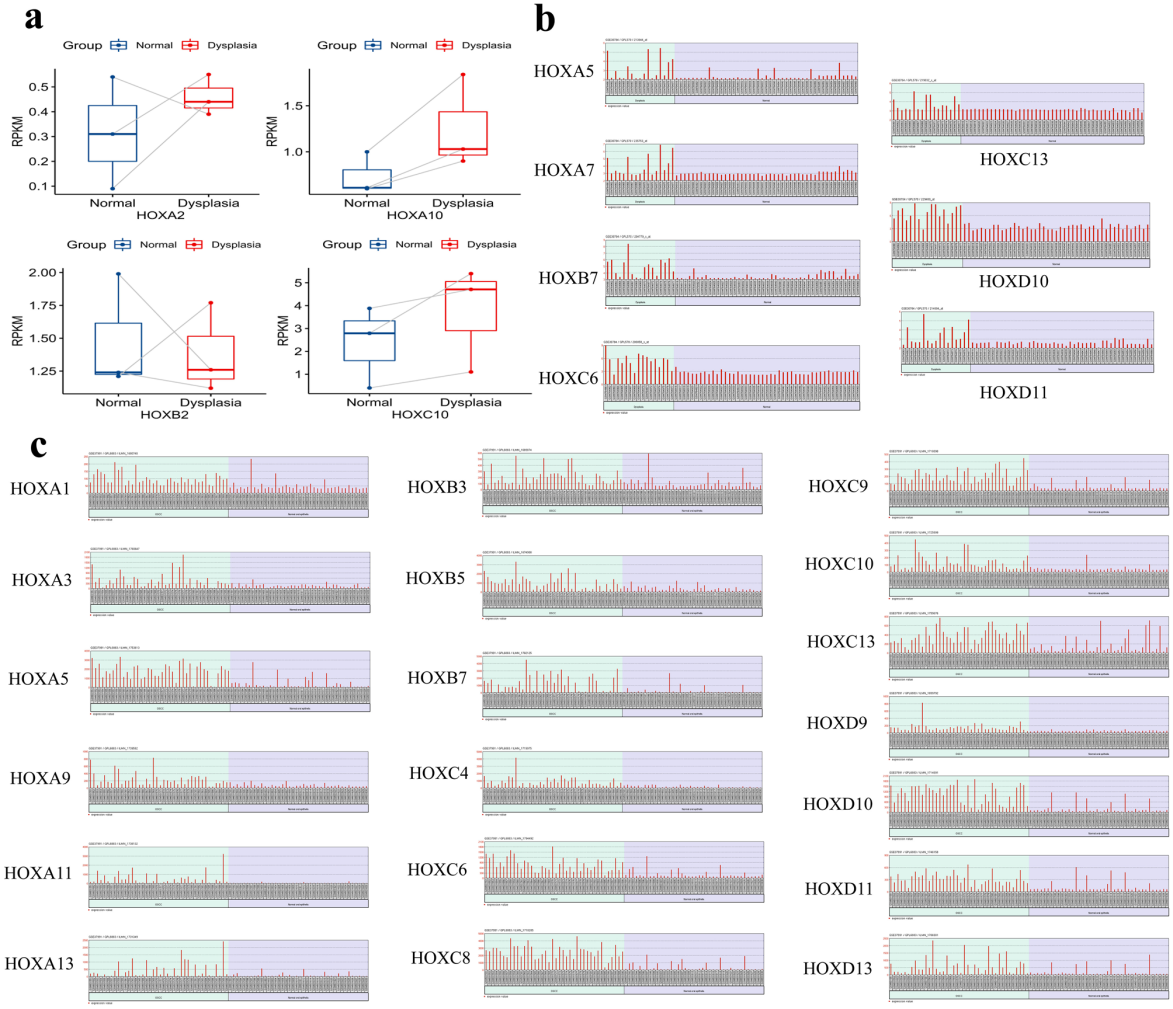
(**a**–**c**): *HOX* genes showed deregulated expression pattern in oral dysplasia compared to the normal. (**a**) *HOXA2*, *HOXA10* and *HOXC10* expression showed an increasing trend whereas *HOXB2* showed a decreased trend of expression during the early onset of dysplasia (n = 3 paired samples). The connected lines represent the paired data points analyzed for each sample. (**b**) *HOXA5*, *HOXA7*, *HOXB7*, *HOXC6, HOXC13*, *HOXD10* and *HOXD11* were shown to be significantly upregulated (*p* < 0.01) in the GSE30784 dataset in a panel of 17 dysplasia and 45 oral mucosa tissues. (**c**) *HOXA1, HOXA3, HOXA5, HOXA9, HOXA11, HOXA13, HOXB3, HOXB5, HOXB7, HOXC4, HOXC6, HOXC8, HOXC9, HOXC10, HOXC13, HOXD10, HOXD11* and *HOXD13* were identified to be significantly upregulated in the cohort of patients who are habituated with the risk factors, betel quid chewing, smoking and alcohol consumption daily. These results implicate the role of external hazards in contributing to oral carcinogenesis.

The differential expression of *HOX* genes, either upregulated or downregulated, through the oral cancer progression, was identified with a cut-off of 1% FDR (Table 1 and Figs. 2, 3). The analyzed *HOX* genes using TCGA-HNSC and the GEO datasets are provided in a supplementary file S1. *HOXA2* expression was upregulated in dysplasia but lost during the tumor progression. The *HOXB2* expression was downregulated in both the dysplasia as well as the primary tumor samples. *HOXA7, HOXA10, HOXB7, HOXC6, HOXC10, HOXD10, HOXD11* showed consistent upregulation in the potentially malignant oral lesions through their progression to oral cancer (Figs. 2a–b and 3a–b), which indicated the posterior prevalence of the *HOX* genes during cancer progression. In addition to these, *HOXB7*, *HOXC6*, *HOXC10*, *HOXD10,* and *HOXD11* were also upregulated in a cohort of the patients who had a history of habits (areca nut chewing, smoking and alcohol consumption) (Fig. 3c). These findings confirmed that the differential expression of *HOX* genes was associated with the progression of oral cancer although the associated risk factors influenced the clinical outcome.

**Table 1:** List of differentially upregulated and downregulated *HOX* genes in a panel of normal, dysplastic and primary tumor through the disease progression of oral cancer.

**Dysplasia versus normal**	
Upregulated *HOX* genes	*HOXA2, HOXA5, HOXA7, HOXA10, HOXB7, HOXC6, HOXC10, HOXC13, HOXD10, HOXD11*
Downregulated *HOX* genes	*HOXB2*
**Tumor versus normal (habituated with risk factors)**	
Upregulated *HOX* genes	*HOXA1, HOXA3, HOXA5, HOXA9, HOXA11, HOXA13, HOXB3, HOXB5, HOXB7, HOXC4, HOXC6, HOXC8, HOXC9, HOXC10, HOXC13, HOXD9, HOXD10, HOXD11, HOXD13*
Downregulated *HOX* genes	*–*
**Primary tumor versus normal**	
Upregulated *HOX* genes	*HOXA1, HOXA6, HOXA7, HOXA10, HOXA11, HOXA13, HOXB7, HOXB9, HOXC4, HOXC6. HOXC8, HOXC9, HOXC10, HOXC11, HOXD10, HOXD11, HOXD13*
Downregulated *HOX* genes	*HOXA2, HOXB2, HOXB4*

### HOX gene expression in oral cancer cell lines

**Figure 4:**
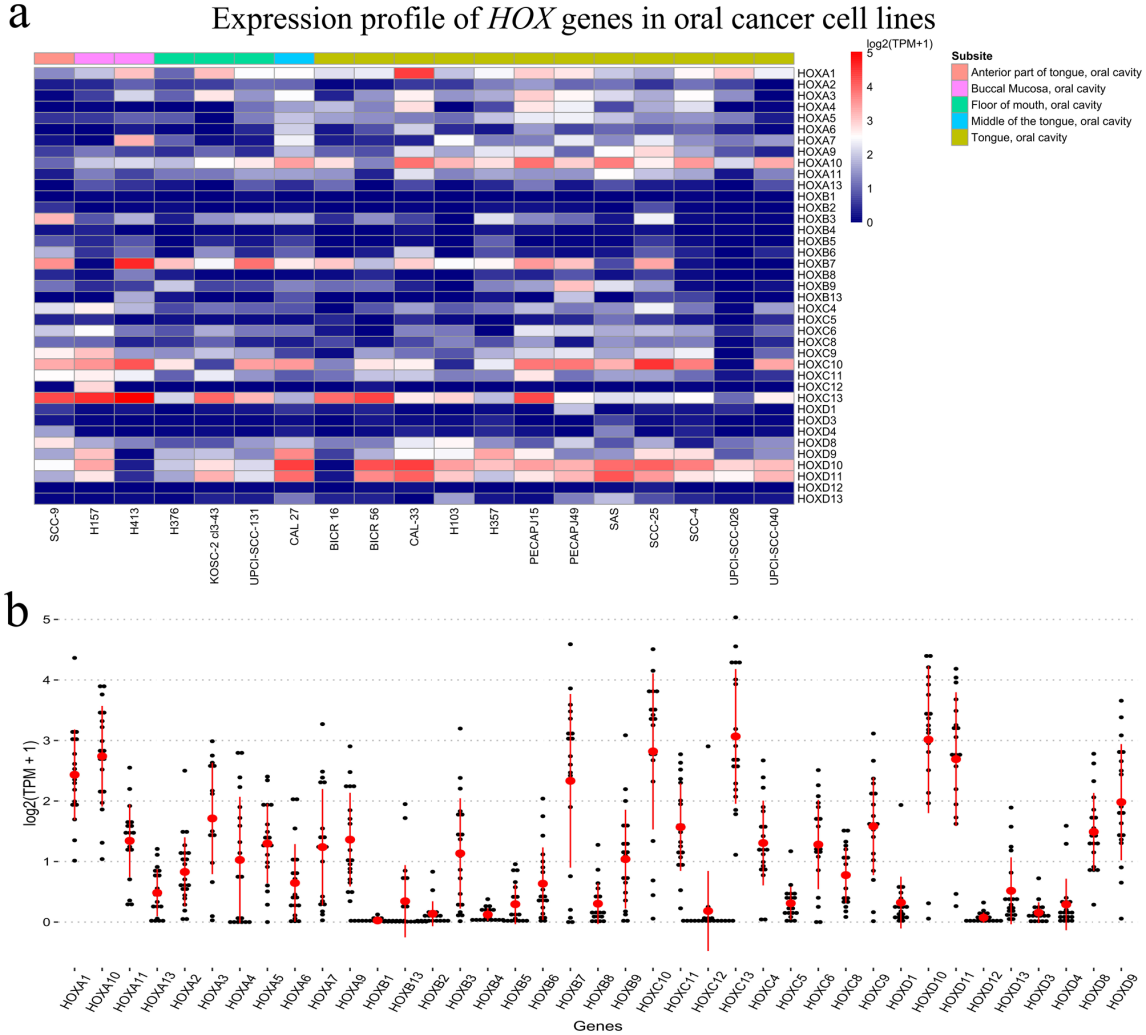
(**a**) Illustrative heatmap depicting the expression level of *HOX* genes expressed as a log2 (TPM + 1) in a panel of 19 oral cancer cell lines screened. The expression states of *HOX* genes have been noted to be modestly varied across the cell lines which are in concordance with the clinical data alluding to their biological origin and genomic variation. (**b**) The pooled mean average of the *HOX* genes in the cell lines is more or less alike to the differentially expressed *HOX* genes analyzed from the panel of patient tissues.

The profile of *HOX* gene expression in oral cancer cell lines specific to their site of origin is provided as Supplementary File S2. The expression of *HOXA1, HOXA7, HOXA10, HOXB7, HOXB9, HOXC4, HOXC6, HOXC9, HOXC10, HOXD10* and *HOXD11* was consistent in both the primary tumor samples from the tongue as well as cell lines, PECAPJ15 (RRID: CVCL_2678), PECAPJ49 (RRID: CVCL_2681), SCC-25 (RRID: CVCL_1682). Likewise, the buccal mucosa-derived H157 (RRID: CVCL_2468) cancer cell lines showed overexpression of *HOXA10, HOXC6, HOXC9, HOXC10* and *HOXD11* which was consistent with the cancer of the buccal mucosa. Further, *HOXA1, HOXA10, HOXB7, HOXB9, HOXC6, HOXC9, HOXC10, HOXD10* and *HOXD11* showed similar expression patterns in both the primary tumor-derived mouth neoplasms and the cell lines, with some variation noted for *HOXB9* in H376 (RRID: CVCL_2463), and *HOXC6* with respect to UPCI-SCC-131 (CVCL_2229) cell lines. The downregulated *HOXB2* and *HOXB4* were consistent across all the 19 cell lines screened. However, the expression patterns were modestly diverse across each cell line screen. This was perhaps due to the cancer cell heterogeneity with respect to the primary site of the tumor and its biological behavior. However, the average expression profile of the *HOX* genes in the cell lines when pooled together was similar to those of the patients’ tissue panel validated experimentally (Figs. 2a–b and 4a–b; Supplementary File S1 & S2).

### HOX genes as developmental cues in oral carcinogenesis

**Figure 5:**
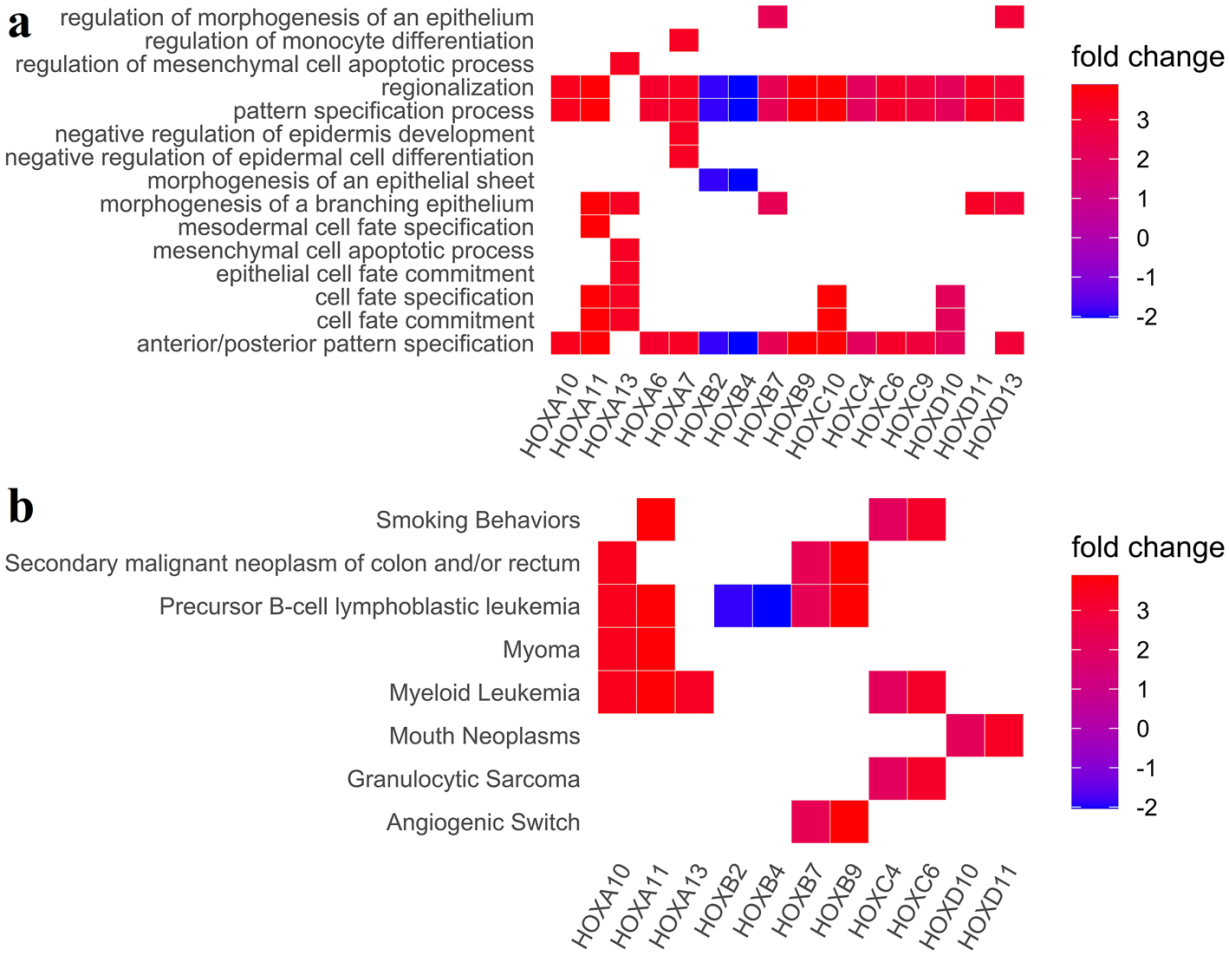
(**a**–**b**): Overrepresentation analysis of differentially expressed *HOX* genes using (**a**) GO biological processes and (**b**) disease ontology (DO). *HOXA11*, *HOXC4* and *HOXC6* have been noted to be associated with smoking. *HOXB7* and *HOXB9* may regulate the angiogenesis whereas *HOXD10*, which regulates the cell fate commitment and specification and *HOXD11*, are noted to be associated with the mouth neoplasm. *HOXA7* and *HOXB2* were involved in the epithelial morphogenesis whose expression was deregulated in the oral carcinoma which implicates the altered expression states of these *HOX* genes contribute to the downstream effects.

The differentially expressed *HOX* genes had hits against the developmentally related events such as epithelial morphogenesis, monocyte differentiation including cell fate specification, and commitment (Fig. 5a). Any alteration in the functional state had consequences leading to phenotypic abnormalities. *HOXA10* participated in the transcriptional misregulation in cancer (Table 2) influencing upstream and downstream target interactions as reported previously^41^. *HOXB9* was involved in the regulation of the BTG (B-cell translocation gene 1–4) family of proteins which functions in the cell cycle mediated events during cancer progression^42^ and was also noted to function as an angiogenic switch along with *HOXB7* (Fig. 5b). Two other posterior *HOX* genes, *HOXD10*, and *HOXD11* were shown to be associated with the mouth neoplasm based on the semantic analysis performed using DO (Fig. 5b). *HOXA11*, *HOXC4* and *HOXC6* (Fig. 5b) expression was upregulated in patients with positive habit history (Fig. 3c).

**Table 2:** *HOX* genes involved in the over represented pathways using integrated molecular pathway level analysis (ImPaLa) tool.

Pathway	Source	Genes involved	*p*-value
Differentiation of white and brown adipocyte	Wikipathways	*HOXC8; HOXC9*	0.000181
btg family proteins and cell cycle regulation	BioCarta	*HOXB9*	0.00735
Transcriptional misregulation in cancer	KEGG	*HOXA10; HOXA11*	0.0102
Signaling events mediated by HDAC Class III	PID	*HOXA10*	0.0315
Keratinocyte differentiation	BioCarta	*HOXA7*	0.0426

### HOX showed interactions with notable key regulators

**Figure 6:**
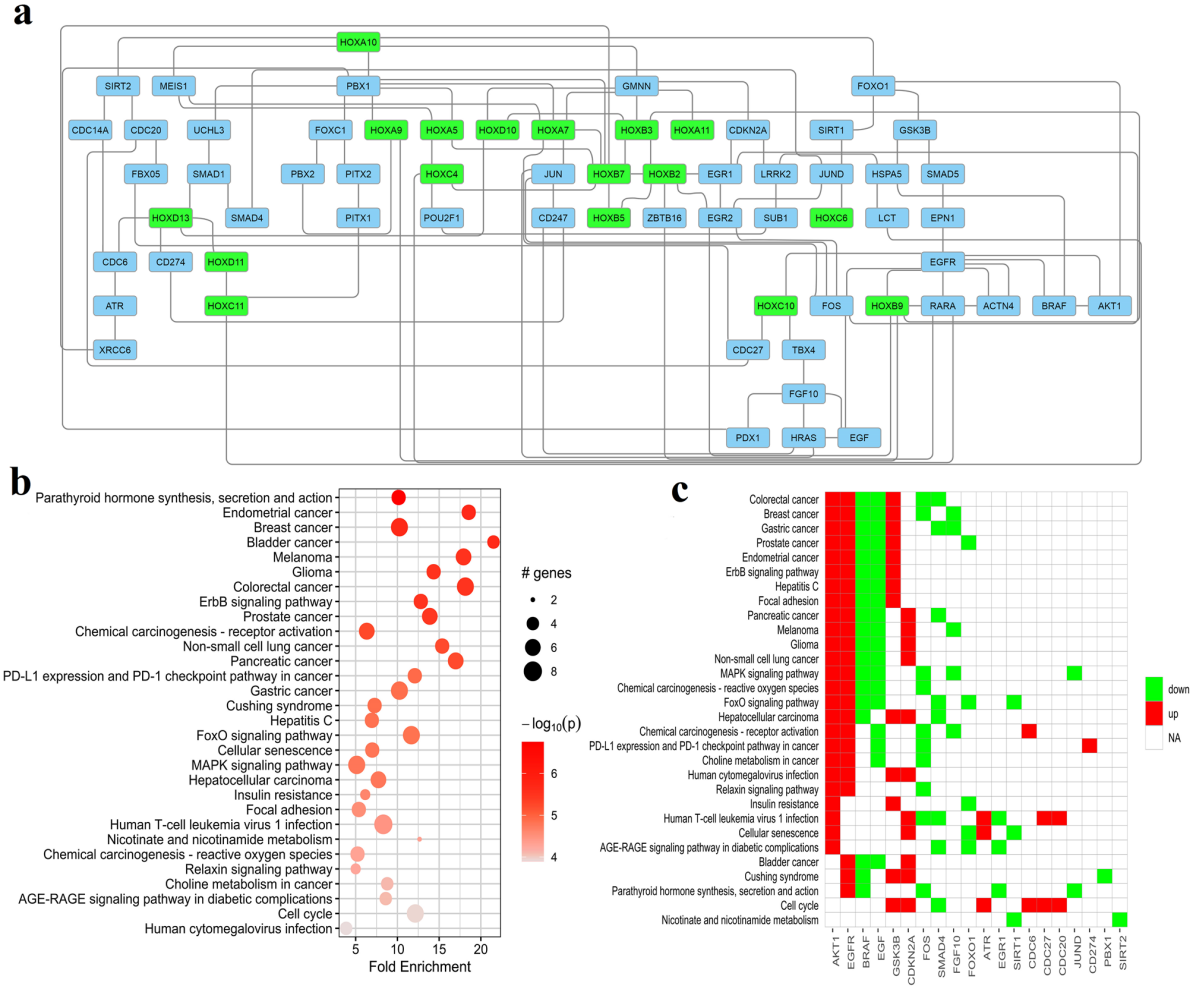
(**a**–**c**): (**a**) HOX subnetwork analysis depicting the protein interactions of HOX downstream to the key cell cycle regulators (*GMNN*, *CDKN2A*, *CDC* group of genes), proto-oncogenes such as *HRAS*, *BRAF*, *JUN* and *PD-L1*. (**b**) Bubble chart depicting the fold enrichment of HOX associated subnetwork query using the KEGG pathways^26–28^ and (**c**) the heatmap illustration of alterations induced by the *HOX*-interacting proteins as noted in the KEGG pathways. The top 20 hits were displayed. These results implicate that the downstream effects of *HOX* contribute to the various alterations in the cancer signaling pathways.

*HOX* genes subnetwork analysis showed crucial interactions with cell cycle regulators such as Geminin (*GMNN*), *CDKN2A* (cyclin-dependent kinase inhibitor 2A), cell division cycle (*CDC*) associated genes such as *CDC6*, *14A*, *20*, *27*, transcriptional regulators like *TBX4* (T-box transcription factor 4), *FOXO1* (Forkhead box protein O1), *FOXC1* (Forkhead box C1), *EGR* (early growth response), *RARA* (retinoic acid receptor alpha), proto-oncogenes such as *JUN*, *HRAS*, *BRAF*, markers such as *PD*-*L1* (programmed cell death ligand 1), *SMAD* family, *FGF10* and other homeobox families of genes such as *POU2F1, PITX2, PDX1, MEIS,* and *PBX* (Fig. 6a). The downstream functional KEGG pathway^26–28^ enrichment analysis of *HOX* interacting subnetwork gene set revealed their involvement in various cancer-related pathways (Fig. 6b) and the differential expression of these genes in KEGG pathways was also identified (Fig. 6c). These interactions pave the way for future studies to decipher cancer-related pathways that are involved by the effect of *HOX* gene downstream activity in oral cancer.

### HOX gene deregulation contributes to the oral carcinoma phenotype

**Figure 7:**
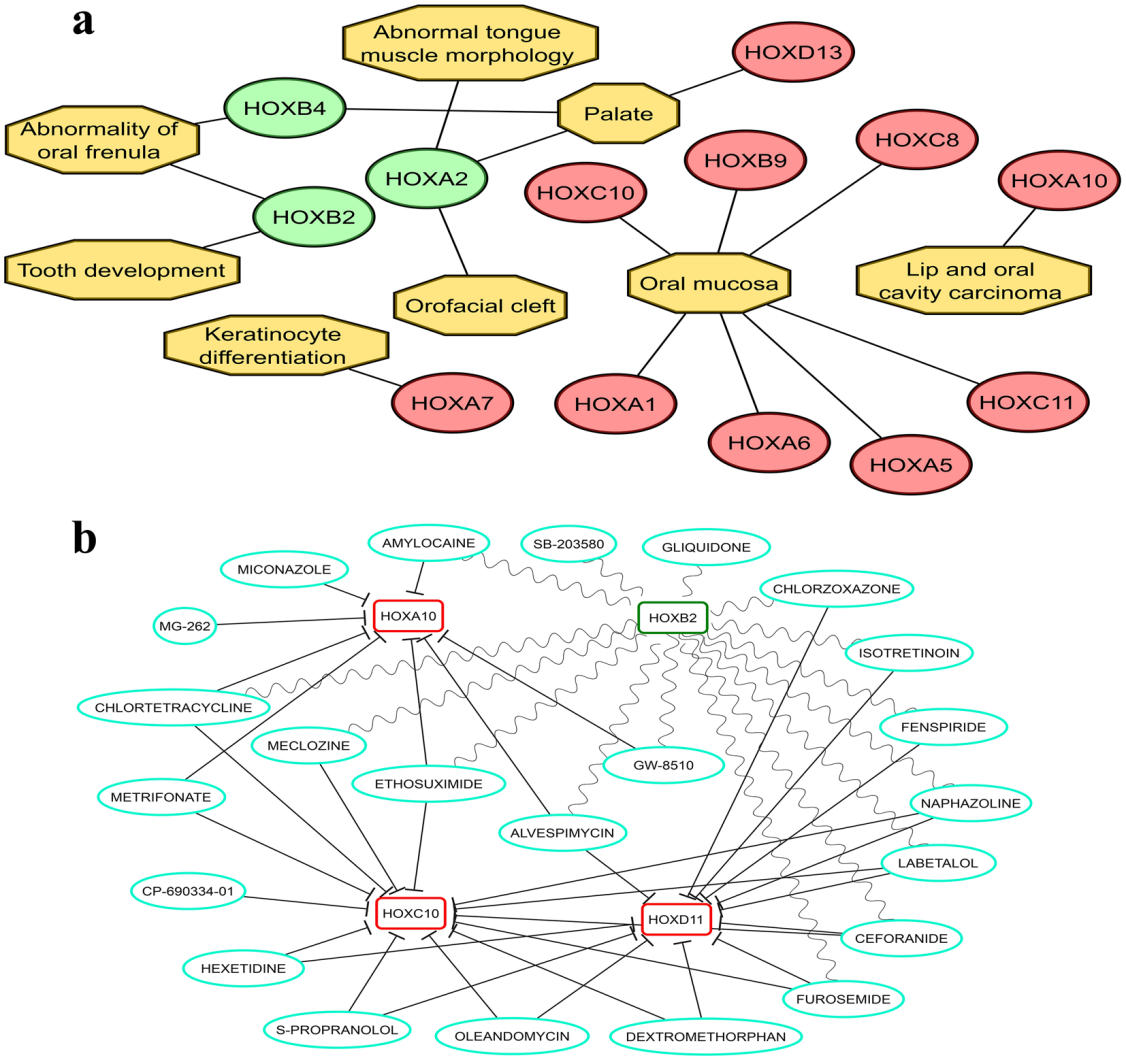
(**a**–**b**): (**a**) Disease-phenotype association of *HOX* genes pertaining to oral cancer. *HOXA1*, *HOXA5*, *HOXA6*, *HOXB9*, *HOXC8*, *HOXC10* and *HOXC1l* were observed to be associated with the oral mucosa whereas *HOXA10* was noted to be involved in the lip and oral cavity carcinoma phenotype. *HOXA2*, *HOXB2*, *HOXB4* and *HOXD13* were associated with the other oral cavity subsites whereas *HOXA7* was noted to be involved in the keratinocytes differentiation phenotype. Upregulated *HOX* genes in oral cancer are represented in red color whereas downregulated *HOX* genes are represented in green color. (**b**) Drug gene interaction network computed using CMap drug rank matrix to identify the potential therapeutic targets. The interaction of drugs that represses the regulation of *HOXA10*, *HOXC10* and *HOXD11* was represented with an inhibition line type arrow whereas the drug that promotes the activity of *HOXB2* was represented with a curved arrow.

Based on the phenotype analysis of differentially expressed *HOX* genes (Figs. 2, 3 and 7a), the upregulated *HOXA1, HOXA6. HOXA5, HOXB9, HOXC8, HOXC10, and HOXC11* genes were specific to certain sites of the oral cavity whereas *HOXA10* was associated with the development of the carcinoma of the lip and oral cavity. The downregulated *HOXA2*, *HOXB2*, *HOXB4* and the upregulated *HOXD13* were associated with morphological changes influenced by specific oral cavity subsites whereas upregulated *HOXA7* was shown to be involved in the keratinocytes differentiation phenotype. These findings indicate that deregulation in the expression of homeobox genes contributes to the carcinoma phenotype as an effect of disruption in developmental coordination during the onset of oncogenesis. This reflects the need for normal expression of these *HOX* genes localized to their developmental sites (Fig. 7a). These findings indicate that the role of *HOX* genes in oral carcinogenesis is tightly modulated and transcriptionally active as previously reported^41^.

### Identification of HOX proteins as potential therapeutic targets

*HOXB2*, *HOXA10*, *HOXC10* and *HOXD11* were noted to be the central hub *HOX* genes involved in promoting oral tumor phenotype (Fig. 7a) amenable to the antagonistic effect of the drugs (Fig. 7b). The wavy arrows indicate the drugs that upregulate the target genes whereas the inhibitory arrows are the drugs that downregulate the target genes. The queried drug-gene interactions (*p* < 0.05) could potentially be used as a part of a chemotherapy regime to reverse the phenotype abnormalities associated with the oral tumor development driven by the deregulation of *HOX* genes. The mechanistic action and insights into the queried drugs reported are discussed further.

## Discussion

*HOX* genes are well established in specifying the developmental states whose upregulation or downregulation has been noted to be involved in carcinogenesis^6,7^. Our analysis showed that these deregulated *HOX* genes which have been described as being involved in developmental aberrations could lead to morphogenetic changes during oral carcinogenesis. Moreover, *HOX* genes are epigenetically regulated via DNA methylation and altered histone modifications, which when dysregulated could potentially be driving the normal cell toward the neoplastic phenotype^13,43^.

The occurrence of oral squamous cell carcinoma has been characterized as a step-wise process in which the normal oral healthy mucosa, after prolonged carcinogenic influences, undergoes a series of changes to develop into the primary invasive tumor^44^. Some studies have shown that *HOXA10* functions in the regulation of proliferation, migration, and invasion and has been reported as a less aggressive tumor phenotype^10^ whereas *HOXC10,* which regulates oral tumorigenesis through Wnt-EMT signaling pathways, might play a pivotal role in metastasis of OSCC (oral squamous cell carcinoma) as studied in an in vivo xenograft model^11^. *HOXD10* was hypermethylated in oral squamous cell carcinoma^45^, and its expression varied across the different stages of tumor progression inducing invasion and causing reciprocal effects with its knockdown in oral cancer cell lines^46^. However, epigenetic regulation of *HOXD10* influencing the steady-state gene expression is yet to explore. *HOXA* cluster genes showed consistent overexpression in OSCC compared to the normal oral healthy mucosal tissues. In particular, *HOXA1* overexpression was correlated with a poor prognosis^47^.

In our analysis, *HOXA2, HOXA5, HOXA7, HOXA10, HOXB2, HOXB7, HOXC6, HOXC10, HOXC13, HOXD10,* and *HOXD11* were differentially expressed in potentially malignant oral dysplastic lesions compared to the normal oral mucosa indicating their early involvement in the malignant transformation. Of particular significance is the posterior prevalence with 5′ *HOX* genes increasingly expressed in the development of oral cancer phenotype. While *HOXA2* was upregulated in dysplasia but lost during the development of oral cancer, HOXB2 expression was downregulated in both dysplasia and primary tumor. Similar findings were reported that *HOXB2* and *HOXB4* were downregulated in OSCC^13,48^ whereas *HOXB7* expression which correlated positively with the cell proliferation was associated with a poor prognosis. Further, H3K9me3 (tri-methylation at the 9th lysine residue of Histone H3 protein) marks associated with the heterochromatin state formation were present at higher levels on *HOXB7*, *HOXC10*, *HOXC13,* and *HOXD8* in normal oral keratinocytes (OKF6-TERT1R) compared to the tongue derived squamous cell carcinoma-9 (SCC-9) cells^43^. These findings indicate that epigenetic machinery modulates the activity of *HOX* genes in oral cancer which is yet to be fully explored.

*HOX* genes expression concerning oral cancer phenotype was noted to be varied among the subset of *HOX* genes screened. This could be attributable to the tumor heterogeneity and tissue specificity of *HOX* genes^6,7^ causing locoregional aberrations during tumor development. Of the *HOX* genes analyzed, *HOXA1, HOXA2, HOXA5, HOXA6, HOXA10, HOXB2, HOXB4, HOXB9, HOXC6, HOXC8, HOXC10,* and *HOXC11* have noted phenotype associations of the oral cavity. This confirms the reason for morphological alterations noted in the oral cavity during tumorigenesis was due to altered *HOX* gene expression.

Further, the subnetwork driven regulatory analysis showed *HOX* genes interactions with the cell cycle regulators (*GMNN*, *CDKN2A*, *CDC* associated proteins), other families of homeobox genes (*TBX4*, *FOXO1*, *FOXC1*, *MEIS*, *PBX*, *POU2F1*) and the proto-oncogenes (*JUN*, *HRAS*, *BRAF*) leading to the alterations of various cancer related signaling pathways. It was reported that POU2F1, identified through the subnetwork analysis, regulates both *HOXD10* and *HOXD11* activity which drives the proliferative and invasive phenotype^49^. Deciphering the molecular interactions of *HOX* genes acting downstream may provide crucial insight into the regulatory network of oral carcinogenesis.

Cell lines are crucial for performing the in vitro validation of specific cancer-associated genes, which could be potential diagnostic markers and therapeutic targets. The choice of a suitable cell line with a careful interpretation of the clinical data is necessary to derive clinically significant results from the in vitro work. In addition to the clinical studies and descriptive studies of samples, assumptions derived about cancer can be tested in cell lines of interest, which are originally isolated from carcinoma patients and also from transgenic models via genetic manipulation. To understand the key *HOX* genes among the cluster that are deregulated from their normal regulation patterns during oral cancer disease progression, we selected 19 oral cancer cell lines based on their characteristic features for screening *HOX* expression patterns in comparison to the patient samples. These analyses are particularly beneficial in identifying a suitable model to study specific HOX or a group of HOX genes.

Among the drugs screened to reverse the acquired phenotype, MG-262 (Z-Leu-Leu-Leu-B(OH)2), a proteasome inhibitor, was reported to be involved in cell growth arrest by promoting the expression of cell cycle inhibitors (p21 and p27), and by driving cell death through the activation of mitogen-activated protein kinase phosphatase 1 (MAPK1) and c-Jun phosphorylation^50^. Chlorzoxazone, a relaxant, was reported to function in the alteration of Ca^2+^ signaling and cell viability by promoting Ca^2+^ independent cell death in human oral cancer cells^51^. A study^52^ reported that low doses of isotretinoin (13-cis retinoic acid) resulted in the regression of potentially malignant oral lesions. Furosemide, a modulator of cellular pumps, was potentially found to be an effective therapeutic regimen that reverses the multidrug resistance in cancer cells and could be used as an adjunct in cancer therapy^53^. S-Propranolol (PRO), a non-selective beta-adrenergic receptor antagonist (beta-blocker) could be a novel adjunctive treatment for HNSCC as it has been shown to inhibit proliferation, invasion, and angiogenesis, and modulate tumor cell sensitivity^54^. Alvespimycin was reported to function as an antitumor agent which sequestrates the target proteins mediated through HSP90 inhibition directing selective proteasomal degradation of BRAF, a proto-oncogene^55^. GW-8510, also belonging to the class of antineoplastics, acts as a cyclin-dependent kinase inhibitor^56^. These repurposed drugs queried from the CMap dataset could be used as adjuncts of the therapeutic regimen and/or for targeted therapy of *HOX* genes studied.

## Conclusion

The posterior prevalent 5′ *HOX* genes were expressed more commonly in oral tumor samples compared to the anterior *HOX* genes, reflecting the loss of expression of anterior *HOX* genes upon the onset of tumorigenesis and deregulation of temporospatial patterning. The development of suitable orthotopic models targeting the specific *HOX* genes identified here would help facilitate future functional studies. Several *HOX* genes, including *HOXA10*, *HOXB2, HOXC10*, and *HOXD11*, could serve as potential therapeutic targets, which would reverse the oral tumor phenotype.

## Supplementary Information

Supplementary Information 1.

Supplementary Information 2.

## Data Availability

All the data used in the present study are freely available for the research community to access from publicly archived datasets, analyzed and/ generated from DepMap repository (https://depmap.org/portal/download/), Genomic Data Commons portal (https://portal.gdc.cancer.gov/), Gene Expression Omnibus Datasets ((https://www.ncbi.nlm.nih.gov/geo/)—GSE72627, GSE30784, GSE37991).

## Code availability

All source codes used are publicly available on GitHub for the research community to access and reproduce the data using DepMap (https://github.com/broadinstitute/depmap_omics), ClusterProfiler (https://github.com/YuLab-SMU/clusterProfiler), pathfinder (https://github.com/egeulgen/pathfindR), Dr. Insight (https://github.com/cran/DrInsight) and Cytoscape (https://github.com/cytoscape/cytoscape.js).
