# Supplementary material for: Identification of HOX signatures contributing to oral cancer phenotype
Source: Sci Rep. 2022 Jun 16;12:10123. doi: 10.1038/s41598-022-14412-6 (PMC9203786; doi:10.1038/s41598-022-14412-6)
Supplement: Supplementary file 1 — Supplementary Information 1. [file 41598_2022_14412_MOESM1_ESM.docx]

**Supplementary File S1**

**Table S1:** List of differentially expressed significant HOX genes in TCGA-HNSC derived oral cancer dataset analyzed using DESeq2 R package.

| Primary tumor (n=234) vs normal (n=18) – oral cavity cancer | | | | | | |
| --- | --- | --- | --- | --- | --- | --- |
| Symbol | baseMean | log2FoldChange | lfcSE | stat | pvalue | Padj |
| *HOXD11* | 259.3117 | 3.477037 | 0.365083 | 6.784857 | 1.16E-11 | 4.53E-10 |
| *HOXA10* | 142.981 | 3.295288 | 0.374858 | 6.12308 | 9.18E-10 | 1.79E-08 |
| *HOXC10* | 234.5605 | 3.528377 | 0.467217 | 5.411565 | 6.25E-08 | 8.12E-07 |
| *HOXC11* | 132.2445 | 2.968551 | 0.368558 | 5.341218 | 9.23E-08 | 9.00E-07 |
| *HOXC8* | 55.13211 | 3.29015 | 0.465031 | 4.924729 | 8.45E-07 | 6.59E-06 |
| *HOXA11* | 45.25415 | 3.76228 | 0.565676 | 4.88315 | 1.04E-06 | 6.59E-06 |
| *HOXC6* | 86.29672 | 2.655908 | 0.340831 | 4.858439 | 1.18E-06 | 6.59E-06 |
| *HOXA1* | 235.5179 | 2.314562 | 0.297257 | 4.422306 | 9.77E-06 | 4.76E-05 |
| *HOXB9* | 70.5904 | 3.532349 | 0.599227 | 4.226029 | 2.38E-05 | 0.000103 |
| *HOXB2* | 110.8972 | -2.16045 | 0.29094 | -3.98862 | 6.65E-05 | 0.000236 |
| *HOXC9* | 76.06822 | 2.51144 | 0.377078 | 4.0083 | 6.12E-05 | 0.000236 |
| *HOXB4* | 40.47111 | -2.35152 | 0.343636 | -3.933 | 8.39E-05 | 0.000273 |
| *HOXD10* | 443.7136 | 2.23671 | 0.318595 | 3.881764 | 0.000104 | 0.000311 |
| *HOXA7* | 38.60263 | 3.262797 | 0.591166 | 3.827683 | 0.000129 | 0.00036 |
| *HOXD13* | 171.4831 | 2.877012 | 0.507836 | 3.696102 | 0.000219 | 0.000569 |
| *HOXA13* | 32.23207 | 3.159987 | 0.628672 | 3.435792 | 0.000591 | 0.001398 |
| *HOXB7* | 319.0396 | 2.29152 | 0.376827 | 3.427356 | 0.000609 | 0.001398 |
| *HOXA6* | 12.74502 | 2.970698 | 0.586235 | 3.361618 | 0.000775 | 0.001679 |
| *HOXA2* | 23.65583 | -1.97455 | 0.332083 | -2.93467 | 0.003339 | 0.006854 |
| *HOXC4* | 99.54981 | 1.856696 | 0.32701 | 2.619789 | 0.008798 | 0.017157 |
| Cheek mucosa derived primary tumor (n=19) vs normal (n=18) | | | | | | |
| *HOXC10* | 109.7908 | 4.182311 | 0.591121 | 5.383516 | 7.30E-08 | 2.85E-06 |
| *HOXA10* | 38.51258 | 3.197979 | 0.49338 | 4.454943 | 8.39E-06 | 0.000164 |
| *HOXA11* | 14.59231 | 3.7715 | 0.649894 | 4.264541 | 2.00E-05 | 0.00026 |
| *HOXB9* | 31.02284 | 4.170199 | 0.757237 | 4.186538 | 2.83E-05 | 0.000276 |
| *HOXB7* | 149.5549 | 2.763012 | 0.48079 | 3.666905 | 0.000246 | 0.001915 |
| *HOXC6* | 27.71997 | 2.61351 | 0.472486 | 3.414938 | 0.000638 | 0.004147 |
| *HOXA13* | 12.40898 | 3.623969 | 0.788081 | 3.329566 | 0.00087 | 0.00424 |
| *HOXC9* | 27.52442 | 2.608851 | 0.479624 | 3.354401 | 0.000795 | 0.00424 |
| *HOXD11* | 61.3357 | 2.962587 | 0.629069 | 3.119828 | 0.00181 | 0.007841 |
| *HOXA2* | 19.73687 | -2.4355 | 0.493194 | -2.91061 | 0.003607 | 0.014068 |
| Gingiva derived primary tumor (n=11) vs normal (n=18) | | | | | | |
| *HOXB4* | 33.80935 | -3.47447 | 0.534465 | -4.62981 | 3.66E-06 | 7.14E-05 |
| *HOXD11* | 57.04238 | 3.959505 | 0.632023 | 4.682594 | 2.83E-06 | 7.14E-05 |
| *HOXA7* | 12.68644 | 4.361648 | 0.763331 | 4.403917 | 1.06E-05 | 8.29E-05 |
| *HOXA10* | 26.03041 | 3.587681 | 0.583356 | 4.435855 | 9.17E-06 | 8.29E-05 |
| *HOXD10* | 113.9561 | 2.952333 | 0.442622 | 4.410836 | 1.03E-05 | 8.29E-05 |
| *HOXA11* | 7.525629 | 3.807252 | 0.803361 | 3.494385 | 0.000475 | 0.002647 |
| *HOXB7* | 84.44335 | 2.867754 | 0.53376 | 3.49924 | 0.000467 | 0.002647 |
| *HOXA6* | 3.54075 | 3.766574 | 0.807934 | 3.424258 | 0.000616 | 0.002777 |
| *HOXC10* | 34.16608 | 3.421039 | 0.709217 | 3.413682 | 0.000641 | 0.002777 |
| *HOXB2* | 90.56685 | -2.63785 | 0.491644 | -3.33138 | 0.000864 | 0.00337 |
| *HOXC6* | 15.43515 | 2.653043 | 0.552606 | 2.991359 | 0.002777 | 0.009847 |
| *HOXA13* | 5.149874 | 3.252082 | 0.769122 | 2.92812 | 0.00341 | 0.011083 |
| Mouth derived primary tumor (n=71) vs normal (n=18) | | | | | | |
| *HOXA10* | 120.3554 | 3.56651 | 0.395925 | 6.48232 | 9.03E-11 | 3.52E-09 |
| *HOXD11* | 233.1107 | 3.373894 | 0.402842 | 5.89287 | 3.80E-09 | 7.40E-08 |
| *HOXB9* | 56.92198 | 3.770001 | 0.57436 | 4.822762 | 1.42E-06 | 1.84E-05 |
| *HOXC10* | 155.8619 | 3.13154 | 0.474432 | 4.492829 | 7.03E-06 | 6.85E-05 |
| *HOXA11* | 31.76076 | 3.418352 | 0.564881 | 4.281174 | 1.86E-05 | 0.000124 |
| *HOXB7* | 266.2924 | 2.415456 | 0.331039 | 4.2758 | 1.90E-05 | 0.000124 |
| *HOXC6* | 56.90353 | 2.317497 | 0.315852 | 4.171247 | 3.03E-05 | 0.000148 |
| *HOXC8* | 41.09424 | 2.981004 | 0.473464 | 4.184063 | 2.86E-05 | 0.000148 |
| *HOXA7* | 28.50904 | 3.302473 | 0.581986 | 3.956233 | 7.61E-05 | 0.00033 |
| *HOXA13* | 26.69336 | 3.329397 | 0.612968 | 3.800196 | 0.000145 | 0.000564 |
| *HOXD10* | 370.3326 | 2.270597 | 0.336507 | 3.775837 | 0.000159 | 0.000565 |
| *HOXC11* | 104.179 | 2.576184 | 0.422955 | 3.726605 | 0.000194 | 0.000631 |
| *HOXD13* | 173.2972 | 2.835437 | 0.497385 | 3.690175 | 0.000224 | 0.000672 |
| *HOXC9* | 68.40591 | 2.449689 | 0.39588 | 3.66194 | 0.00025 | 0.000697 |
| *HOXA6* | 10.34868 | 3.104066 | 0.594358 | 3.540064 | 0.0004 | 0.00104 |
| *HOXA1* | 166.9853 | 2.169847 | 0.342591 | 3.414701 | 0.000639 | 0.001556 |
| *HOXB4* | 39.96337 | -2.11651 | 0.357126 | -3.12637 | 0.00177 | 0.00406 |
| *HOXB2* | 108.8148 | -1.99152 | 0.336899 | -2.9431 | 0.003249 | 0.007041 |
| Tongue derived primary tumor (n=127) vs normal (n=18) | | | | | | |
| *HOXA10* | 137.0948 | 3.833156 | 0.408867 | 6.929278 | 4.23E-12 | 1.65E-10 |
| *HOXC6* | 131.8703 | 3.750853 | 0.447305 | 6.14984 | 7.76E-10 | 1.01E-08 |
| *HOXD11* | 232.3332 | 3.628269 | 0.42682 | 6.15779 | 7.38E-10 | 1.01E-08 |
| *HOXC8* | 57.72184 | 3.866911 | 0.478716 | 5.988746 | 2.11E-09 | 2.06E-08 |
| *HOXC10* | 274.6125 | 4.176233 | 0.549667 | 5.778466 | 7.54E-09 | 5.88E-08 |
| *HOXA1* | 228.2743 | 2.715494 | 0.313914 | 5.464859 | 4.63E-08 | 3.01E-07 |
| *HOXA11* | 41.66915 | 4.038459 | 0.570286 | 5.327959 | 9.93E-08 | 5.53E-07 |
| *HOXC11* | 116.7053 | 3.008819 | 0.404111 | 4.970965 | 6.66E-07 | 3.25E-06 |
| *HOXC9* | 84.77209 | 3.007586 | 0.433515 | 4.630946 | 3.64E-06 | 1.58E-05 |
| *HOXA7* | 35.50989 | 3.655716 | 0.594956 | 4.463721 | 8.05E-06 | 3.14E-05 |
| *HOXB9* | 62.56249 | 3.777121 | 0.663848 | 4.183368 | 2.87E-05 | 0.000102 |
| *HOXD10* | 426.9063 | 2.578759 | 0.379638 | 4.158595 | 3.20E-05 | 0.000104 |
| *HOXC4* | 119.1274 | 2.590893 | 0.387097 | 4.109805 | 3.96E-05 | 0.000119 |
| *HOXA13* | 32.39658 | 3.633211 | 0.651335 | 4.042788 | 5.28E-05 | 0.000147 |
| *HOXA6* | 10.59587 | 3.352722 | 0.599434 | 3.924908 | 8.68E-05 | 0.000226 |
| *HOXB7* | 260.3562 | 2.395091 | 0.41482 | 3.36312 | 0.000771 | 0.001879 |
| *HOXD13* | 141.0925 | 2.738897 | 0.571614 | 3.042081 | 0.002349 | 0.00539 |
| *HOXC5* | 4.40452 | 2.719744 | 0.607611 | 2.830337 | 0.00465 | 0.010075 |

**Table S2:** List of differentially expressed significant HOX genes in a panel of 17 dysplastic and 45 normal oral tissues (GSE30784)

| ID | adj.P.Val | P.Value | T | B | logFC | Gene.symbol |
| --- | --- | --- | --- | --- | --- | --- |
| 229400_at | 4.37E-13 | 4.78E-16 | 1.10E+01 | 26.17377 | 3.2 | *HOXD10* |
| 206858_s_at | 4.48E-12 | 7.28E-15 | 1.02E+01 | 23.53055 | 2.71 | *HOXC6* |
| 204779_s_at | 1.66E-06 | 2.38E-08 | 6.41 | 8.92804 | 1.44 | *HOXB7* |
| 219832_s_at | 2.41E-05 | 5.46E-07 | 5.6 | 5.89065 | 5.87E-01 | *HOXC13* |
| 216973_s_at | 3.97E-05 | 9.82E-07 | 5.44 | 5.32369 | 1.11 | *HOXB7* |
| 214604_at | 3.98E-05 | 9.87E-07 | 5.44 | 5.31875 | 8.46E-01 | *HOXD11* |
| 235753_at | 3.46E-04 | 1.32E-05 | 4.74 | 2.82141 | 9.82E-01 | *HOXA7* |
| 218959_at | 4.89E-04 | 2.01E-05 | 4.62 | 2.41865 | 7.58E-01 | *HOXC10* |
| 207373_at | 9.20E-04 | 4.34E-05 | 4.41 | 1.68112 | 6.32E-01 | *HOXD10* |
| 213844_at | 2.81E-03 | 1.70E-04 | 4.01 | 0.38554 | 7.97E-01 | *HOXA5* |

**Table S3:** List of differentially expressed significant HOX genes in a panel of pairwise 40 normal and tumor patients habituated with areca nut, alcohol and smoking (GSE37991).

| ID | adj.P.Val | P.Value | T | B | logFC | Gene.symbol |
| --- | --- | --- | --- | --- | --- | --- |
| ILMN_1718285 | 1.27E-19 | 4.21E-22 | 13.28853 | 39.85971 | 3.625074 | *HOXC8* |
| ILMN_1714691 | 1.40E-15 | 1.96E-17 | 10.8215 | 29.26275 | 2.607887 | *HOXD10* |
| ILMN_1738132 | 6.99E-15 | 1.15E-16 | 10.4266 | 27.48272 | 2.68609 | *HOXA11* |
| ILMN_1659792 | 1.02E-14 | 1.78E-16 | 10.33056 | 27.02601 | 1.238589 | *HOXD9* |
| ILMN_1794492 | 1.14E-14 | 2.01E-16 | 10.30351 | 26.97414 | 1.961321 | *HOXC6* |
| ILMN_1757184 | 4.63E-14 | 9.76E-16 | 9.954598 | 25.30448 | 1.13744 | *HOXC4* |
| ILMN_1719975 | 8.78E-14 | 2.04E-15 | 9.791882 | 24.69446 | 2.044088 | *HOXC4* |
| ILMN_1753613 | 1.11E-11 | 4.45E-13 | 8.61164 | 19.37682 | 2.615595 | *HOXA5* |
| ILMN_1718898 | 2.07E-11 | 8.80E-13 | 8.462456 | 18.62431 | 1.931242 | *HOXC9* |
| ILMN_1746158 | 5.30E-11 | 2.53E-12 | 8.230946 | 17.66542 | 1.711255 | *HOXD11* |
| ILMN_1739582 | 3.42E-10 | 1.99E-11 | 7.776809 | 15.59141 | 1.506272 | *HOXA9* |
| ILMN_1725899 | 2.28E-08 | 2.16E-09 | 6.732283 | 10.79133 | 1.224896 | *HOXC10* |
| ILMN_1680740 | 3.32E-08 | 3.25E-09 | 6.639158 | 10.32782 | 1.049413 | *HOXA1* |
| ILMN_1702125 | 5.18E-08 | 5.33E-09 | 6.52667 | 10.17345 | 2.365211 | *HOXB7* |
| ILMN_1759676 | 2.19E-07 | 2.68E-08 | 6.155163 | 8.604849 | 1.571164 | *HOXC13* |
| ILMN_1689874 | 3.44E-06 | 5.60E-07 | 5.433736 | 5.59405 | 1.041883 | *HOXB3* |
| ILMN_1674908 | 4.79E-06 | 8.09E-07 | 5.343699 | 5.309047 | 1.251397 | *HOXB5* |
| ILMN_1706901 | 7.37E-06 | 1.30E-06 | 5.227379 | 4.848996 | 1.572901 | *HOXD13* |
| ILMN_1760647 | 2.56E-05 | 5.15E-06 | 4.881623 | 3.54193 | 1.093981 | *HOXA3* |
| ILMN_1731349 | 3.81E-05 | 7.96E-06 | 4.770061 | 2.98781 | 1.664541 | *HOXA13* |
